# Successful Treatment and Secondary Prevention of Venous Thrombosis Secondary to Behçet Disease with Rivaroxaban

**DOI:** 10.1155/2016/2164329

**Published:** 2016-06-29

**Authors:** Ana Boban, Catherine Lambert, Cedric Hermans

**Affiliations:** ^1^Haemostasis and Thrombosis Unit, Division of Haematology, Haemophilia Clinic, Saint-Luc University Hospital, Avenue Hippocrate 10, 1200 Brussels, Belgium; ^2^Division of Haematology, University Hospital Centre Zagreb, Medical School of Zagreb, Kišpatićeva 12, 10000 Zagreb, Croatia

## Abstract

We here present the successful initial treatment and secondary prophylaxis of superficial venous thrombosis secondary to Behçet's disease by a novel anticoagulant drug, rivaroxaban (Xarelto®). To our knowledge, this is the first case of using an oral direct inhibitor of FXa in this setting. Our findings are promising; the outcome was favourable without any adverse effect noted. We propose that the patients with Behçet's disease and venous thrombosis might benefit from the advantages of the new anticoagulant drug.

## 1. Introduction

Behçet disease (BD) is a rare systemic inflammatory disease characterized by exacerbation and remissions. Clinically, the BD presents with recurrent oral and urogenital ulcers, uveitis, skin lesions, and involvement of vascular, gastrointestinal, musculoskeletal, and central nervous system [1]. The etiology of BD is not very well defined yet, although it is known that it involves both genetic and environmental factors. The prevalence of BD is low in the western world; the highest prevalence is seen in Asia, Middle East, and Mediterranean area, which corresponds to the high prevalence of HLA-B51 allele.

Vascular complications are frequent in BD and are most often presented as venous thrombosis (VT) with high risk of recurrence. The largest retrospective study investigating VT in patients with BD found that 36% of patients had VT. Moreover, the incidence of relapse of VT was 36% at 5 years and 55% at 20 years after the first episode of thrombosis [2]. The sites most commonly involved by thrombosis are superficial and common femoral veins [3].

Predisposition for thrombotic tendency in BD is not clear yet. Two mechanisms have been proposed, vasculitis and hypercoagulable status, although several studies failed to demonstrate association between coagulation abnormalities and VT in BD [4]. Still, no agreement was reached regarding the treatment or secondary prophylaxis of VT associated with BD.

We here present the case of a patient with BD and recurrent superficial venous thrombosis treated with oral direct factor Xa inhibitor rivaroxaban (Xarelto).

## 2. Case Report

An 18-year-old male patient was referred to our clinic after a second episode of superficial venous thrombophlebitis of the lower limbs. Diagnosis of venous thrombosis was made few days earlier at the Emergency Department, after the patient complained of having pain and swelling of both legs. Diagnosis of VT was confirmed by elevated level of D-Dimers (851 ng/mL, normal values <500) and ultrasound signs of thrombosis involving internal saphenous vein on the right and the external saphenous vein on the left leg. The treatment with full-dose low molecular weight heparin (LMWH) was started immediately.

The patient was Moroccan; his parents affirmed consanguinity. His father had diabetes and died recently after a cerebrovascular accident. The patient had four sisters, two of whom have diabetes, and two healthy brothers. Family history was negative for Behçet's disease and thromboembolic events.

The patient had a history of recurrent painful ulcers on buccal and lingual mucosal surface, occurring approximately once weekly for years. The first episode of superficial venous thrombosis on the right lower leg occurred two months before and was treated with LMWH for a month, full dose for a week and half dose afterwards, with good resolution of the clot, as estimated clinically. A month later, after an episode of orchitis, the patient was diagnosed with Behçet's disease. The diagnosis was made based on typical clinical symptoms. Colchicine was then introduced into the therapy, with a daily dose of 1 mg.

When we examined the patient, he was taking full-dose enoxaparin for a week and colchicine for a month. He had no pain or swelling of the legs and physical examination showed no signs of thrombosis. We decided that the patient was candidate for a long-term therapy and chose rivaroxaban as the anticoagulant drug of choice. The starting dose was 20 mg daily but was shortly afterwards reduced to 15 mg a day. The patient tolerated rivaroxaban very well. He had no bleeding symptoms and reported no side effects. Moreover, following treatment with colchicine, no new episodes of oral ulcerations were reported. In the follow-up period of 33 months, since the treatment started, the patient did not have any symptoms of BD or recurrent VT.

## 3. Discussion

We here present the case of a patient with BD and recurrent VT successfully treated with direct Xa inhibitor. To our knowledge, this is the first case of BD associated VT treated with rivaroxaban.

Venous thromboembolism plays an important role in the course of BD. The incidence of VT among patients with BD is high, as well as the risk of its recurrence. Besides, the mortality in patients with BD is in significant part associated with TV, usually related to thrombosis of vena cava or pulmonary embolism [5]. Hence, the patients with BD bear a high risk of a life-threatening thrombotic occlusion. On the other hand, no consensus has been made on guidelines for optimal antithrombotic treatment and secondary prevention of venous thromboembolism in BD. Since there are no large randomised studies addressing this question, decisions on therapy of thrombotic complications in BD are mostly based on retrospective cohort studies and case series. According to the published data, VT in the majority of patients with BD was treated with heparin followed by oral anticoagulants, together with immunosuppressive therapy [3]. Immunosuppressive therapy seems to have an important role in the treatment of VT secondary to BD, as the results of retrospective study showed that anticoagulation treatment alone does not protect patients from the recurrence of VT [3].

Our patient experienced the second episode of VT shortly after the first one, while he had no other symptoms of BD during therapy with colchicine. That encouraged us to initialize a long-term anticoagulation therapy and not immunosuppressive therapy. We choose a novel oral direct inhibitor of coagulation factor Xa, rivaroxaban, because of its advantages, fixed daily dose, no need for laboratory monitoring, and limited number of drug interactions. Besides, in the comparison with vitamin K antagonists (VKA) in treating acute VT, rivaroxaban was demonstrated to have a lower risk for major and nonmajor clinically relevant bleeding [6]. Furthermore, rivaroxaban has been proven to be effective in preventing recurrences of VT with an acceptable risk of bleeding when compared to placebo [7]. Additionally, a long-term treatment with VKA may have other side effects besides bleeding, such as inducement of osteoporosis, as VKA inhibit gamma-carboxylation of osteocalcin, a protein involved in bone mineral turnover [8]. Risk of osteoporosis should be taken into account when planning long-term anticoagulation in a young patient.

The fixed doses of rivaroxaban have been proposed in the previous studies, 15 mg twice daily for acute treatment and 20 mg daily afterwards [7]. However, we decided to continue the long-term therapy in our patient with reduced dose of rivaroxaban. We considered that 15 mg of rivaroxaban daily might be enough for secondary prophylaxis in our patient, and we found that the dose was adequate for the thromboprophylaxis, although the follow-up was rather too short for proposing conclusions.

Although we base our conclusion on report of a single patient with superficial VT, rivaroxaban seems to have the qualities to serve as an anticoagulant drug for treating and secondary prevention of BD associated VT. Certainly, drawing conclusions on a single case of a rare disease where spontaneous remissions have been described is challenging. Nevertheless, this potential of rivaroxaban should be explored in further studies with a larger cohort of patients including also deep VT, although the conduction of large studies is unlikely, as BD is a rare disease.

## References

[1] Sakane T., Takeno M., Suzuki N., Inaba G. (1999). Behcet's disease. *The New England Journal of Medicine*.

[2] Desbois A. C., Wechsler B., Resche-Rigon M. (2012). Immunosuppressants reduce venous thrombosis relapse in Behçet's disease. *Arthritis and Rheumatism*.

[3] Ahn J. K., Lee Y. S., Jeon C. H., Koh E.-M., Cha H.-S. (2008). Treatment of venous thrombosis associated with Behcet's disease: immunosuppressive therapy alone versus immunosuppressive therapy plus anticoagulation. *Clinical Rheumatology*.

[4] Kiraz S., Ertenli I., Öztürk M. A., Haznedarolu I. C., Çelik I., Çalgüneri M. (2002). Pathological haemostasis and ‘prothrombotic state’ in Behçet's disease. *Thrombosis Research*.

[5] Saadoun D., Wechsler B., Desseaux K. (2010). Mortality in Behçet's disease. *Arthritis and Rheumatism*.

[6] Prins M. H., Lensing A. W. A., Bauersachs R. (2013). Oral rivaroxaban versus standard therapy for the treatment of symptomatic venous thromboembolism: a pooled analysis of the EINSTEIN-DVT and PE randomized studies. *Thrombosis Journal*.

[7] Bauersachs R., Berkowitz S. D., Brenner B. (2010). Oral rivaroxaban for symptomatic venous thromboembolism. *The New England Journal of Medicine*.

[8] Fusaro M., Crepaldi G., Maggi S. (2011). Bleeding, vertebral fractures and vascular calcifications in patients treated with warfarin: hope for lower risks with alternative therapies. *Current Vascular Pharmacology*.

